# REIA: A database for cancer A-to-I RNA editing with interactive analysis

**DOI:** 10.7150/ijbs.69458

**Published:** 2022-03-14

**Authors:** Huimin Zhu, Lu Huang, Songbin Liu, Zhiming Dai, Zhou Songyang, Zhihui Weng, Yuanyan Xiong

**Affiliations:** 1 Key Laboratory of Gene Engineering of the Ministry of Education, Institute of Healthy Aging Research, School of Life Sciences, Sun Yat-sen University, Guangzhou, 510006, China.; 2 School of Automation, Guangdong University of Technology, Guangzhou, 510006, China.; 3 School of Computer Science and Engineering, Sun Yat-sen University, Guangzhou, 510006, China.; 4 Faculty of Health Sciences, University of Macau, Macau, 999078, China.

**Keywords:** A-to-I RNA editing, Cancer, Interactive analysis, Multi-omics, Database

## Abstract

Epitranscriptomic changes caused by adenosine-to-inosine (A-to-I) RNA editing contribute to the pathogenesis of human cancers; however, only a small fraction of the millions editing sites detected so far has clear functionality. To facilitate more in-depth studies on the editing, this paper offers REIA (http://bioinfo-sysu.com/reia), an interactive web server that analyses and visualizes the association between human cancers and A-to-I RNA editing sites (RESs). As a comprehensive database, REIA curates not only 8,447,588 RESs from 9,895 patients across 34 cancers, where 33 are from TCGA and 1 from GEO, but also 13 different types of multi-omic data for the cancers. As an interactive server, REIA provides various options for the user to specify the interested sites, to browse their annotation/editing level/profile in cancer, and to compare the difference in multi-omic features between editing and non-editing groups. From the editing profiles, REIA further detects 658 peptides that are supported by mass spectrum data but not yet covered in any prior works.

## Introduction

RNA editing is one of the most conservative features in RNA evolution. It alters the primary RNA transcripts via insertion, deletion, or base substitution of nucleotides 1. Nearly 90% of human RNA editing is resulted from the adenosine to inosine (A-to-I) conversion at the double-stranded nucleic RNA 2. These A-to-I RNA editing sites (RESs) are key to the pathogenesis of human cancers, as it provides the growth of tumor cells with selective advantages and resistance to apoptosis 3. They also affect many other aspects of cancer, including the expression of cancer-related genes 4, 5, alternative splicing (AS) 6, 7, expression and target of microRNA 8-10, and secondary structure of lncRNA 3. More recently, they were further linked to the change of cancer immune microenvironment 3 and verified to be clinically significant 11, 12.

Despite the importance of these functions, the research community is still lacking a comprehensive understanding on the general functionalities of A-to-I RESs, thus calling for further studies. However, prior works based on the experimental approach often suffer from the loss of genomic information and the risk in off-target edits and delivery 13. In contrast, using the bioinformatic approach to build a database (or web server) and provide functional analysis for A-to-I RNA editing sites (RESs) is more effective in terms of time and cost. Databases of this kind include REDIportal 14, RADAR 15, DARNED 16, and TCEA 17, where the location and annotation information were provided for ten million A-to-I RESs. Although these databases had offered the retrieval of these editing sites, they did not provide a functional analysis for them. The A-to-I editing sites were long known to be closely connected with features of many omics: the A-to-I editing sites have a complementary relationship with the DNA mutation of hepatocellular carcinoma (HCC) risk genes in HCC patients 18; those sites in the 3'UTR region can also affect the miRNA expression in a variety of cancers 19, 20; and they can even be used as the indicator to build a cancer prognostic model 12. Furthermore, the A-to-I RNA editing contributes to the protein diversity of cancer. Such functional non-synonymous editing can affect the structure, the function, and drug targets of the protein, and therefore, it is of great interest to the precise treatment of cancer 21. For these reasons, the association analysis between A-to-I RESs and multi-omic features is essential to the community.

**Figure 1:**
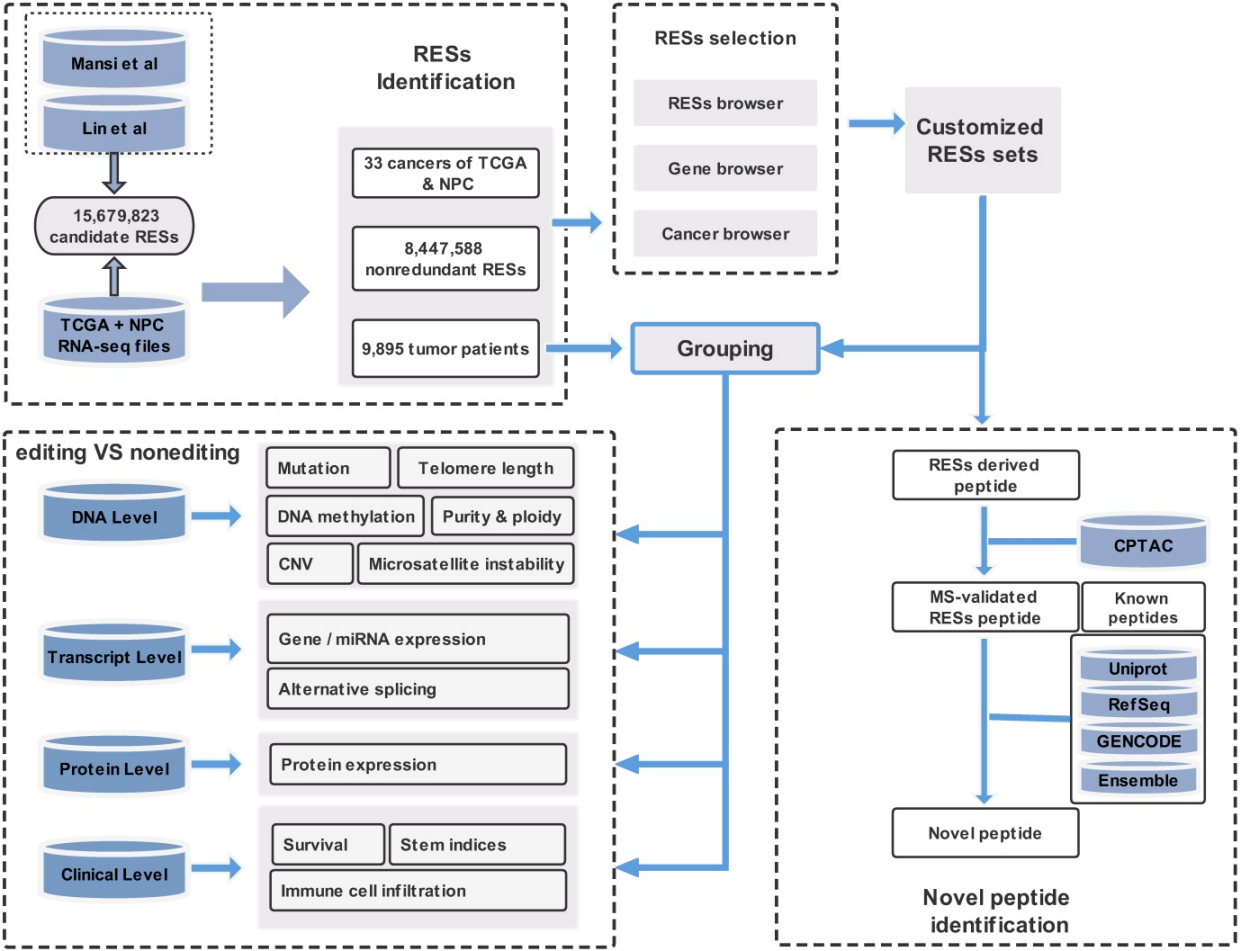
** Overall description of the web server REIA.** Overall description of the web server REIA. REIA has identified 8,447,588 A-to-I RESs from 9,895 patients across 34 cancers (TCGA + GEO), on which 13 different types of multi-omic data are also collected. The editing sites can be retrieved by RES positions, host gene names, cancer types, or any of their combinations. An association analysis between these editing sites and the multi-omic data is provided, which covers 14 aspects of 4 different levels, including DNA, transcript, protein and clinical. Result of the analysis can be downloaded in PDF/PNG/SVG format.

In this paper, we provide a new web server for such analysis. The server is termed REIA, a database for A-to-I RNA editing in cancers with interactive analysis, which is publicly available at http://bioinfo-sysu.com/reia. The architecture of REIA is shown in Figure 1. Different from existing databases that presented only the information stored 14, 17, this new server allows for an iterative analysis, i.e., conditions, such as editing positions and cancer types, can be customized arbitrarily, and their influence on the indices of multi-omic features will be calculated and visualized accordingly. By doing this, the paper contributes these two aspects: (1) In terms of data set, REIA has detected 8,447,588 A-to-I RESs from the RNA-Seq data of 9,895 tumor patients across 34 cancers, where 33 are from TCGA while nasopharyngeal carcinoma (NPC) is from GEO. NPC is a rare tumor of the head and neck, which originates in the nasopharynx. It is more common in southeast Asia and is frequently, but not always, caused by the Epstein-Barr virus (EBV). The detection is carried out using a combination of TCEA 17 and REDIportal 14 to embrace both regular and hyper editing sites. REIA also collects 13 types of multi-omic data related to human cancers from four different levels, namely, DNA level (including somatic mutation, gene copy number, DNA methylation, telomere length, microsatellite instability, tumor purity and ploidy), transcriptome level (including gene expression, miRNA expression, alternative splicing), protein level (protein expression), and clinicopathology level (immune cell infiltration, stem indices, and patient survival). In addition to the above collections, REIA further identifies 658 novel peptides from non-synonymous peptides that are supported by mass spectrum (MS) data. These novel peptides, to the best of the authors' knowledge, have not been covered in any prior reports. As they are cancer-specific, the peptides may affect the occurrence and development of cancers. (2) In terms of analysis, REIA is advantageous to many prior works not only because it is interactive but also because it is multi-functional. On one hand, similar to our previous work, a key enabler for the interactive analysis is the model-view-controller (MVC) framework 22. Unlike prior databases that were mostly using bootstrap 14, 23, REIA here applies MVC to handle user interface, business logic, and data management in a divide-and-conquer fashion, which makes the interaction and maintenance much easier. On the other hand, REIA not only provides a retrieval for the editing sites, but also analyzes and visualizes their association with 13 multi-omics features in 33 different cancers. In addition, it offers for the 658 peptides identified detailed information, such as RES coordinates, sequence, gene name, mutant and wild-type bases on amino acids, codons, and distribution in cancers, paving the way for targeted therapies. The overall architecture of the database is shown in Figure 1.

Summing up, the web server REIA offers a new RNA editing database operating on the per cancer basis and opens up a new avenue for the research in association of A-to-I RNA editing with cancer multi-omics features.

## Materials and Methods

### Detection for A-to-I RNA editing sites

1) *Data Collection:* The overall process is shown in Figure 1. We download the data of 15,679,823 A-to-I editing sites from REDIportal 2.0 14 (hg38, http://srv00.recas.ba.infn.it/atlas/index.html) and merged it with the 8,972,972 sites from TCEA 17 (hg38, http://tcea.tmu.edu.tw). After removing the duplicates, we obtained a candidate set of 15,680,513 sites, which covers most (if not all) editing sites ever detected in human samples, either normal or cancerous. Together we have 9,895 samples, in which 9,697 RNA-seq bam files (V21.0) across 33 cancer types included in TCGA are downloaded from Genomic Data Commons (GDC, https://gdc.cancer.gov), while the remaining 198 files are from GEO (GEO, https://www.ncbi.nlm.nih.gov/geo) on nasopharyngeal carcinoma (NPC). For somatic mutation, we download the data from GDC and for Germline single nucleotide poly-morphisms (SNPs) from GDC and dbSNP (V151, https://www.ncbi.nlm.nih.gov/snp).

2) *RES Detection:* The 15,680,513 sites collected above serves as a candidate set of our RES detection. For mapped reads, we apply REDItools (REDItoolKnown.py) under the same setting as Picardi et al. 24, 25 to detect the A-to-I RESs. For unmapped reads, we apply SPRINT 26 (http://sprint.tianlan.cn), a SNP-free toolkit; however, unlike most previous studies which considered only hyper RESs 14, 17, we detect both hyper and regular RESs from unmapped reads, but leave only those in the candidate set. To further improve the detection quality, we add some filtering similar to 27: supported reads 10, edited sample number per cancer type 2, editing level (defined as edited G reads / A+G reads) 0.1%, and loci annotated only in a single strand. Also, to reduce the false positive rate, we exclude editing sites overlapped with germline SNPs and somatic mutations relevant to DNA variants. Finally, we have detected 110,264,281 regular editing events and 92,751,089 hyper events, from which a comprehensive collection of 8,447,588 A-to-I RESs are identified.

3) *RES Annotation:* To annotate the RESs, we used ANNOVAR 28 (https://annovar.openbioinformatics.org/en/latest) together with Gencode (v34), Refseq and UCSC for gene structure, dbSNP (v151), ExAC, GnomAD (v30) for variant, COSMIC (v92) and ClinVar for disease, RepeatMask for repetitive elements, PhyloP and PhastCons from UCSC (https://genome.ucsc.edu) for conservation score across 100 vertebrates. Annotated sites are then divided either into 8 types by their locations in the genomic region, or into 3 types by the repeat region. The 8 genomic- region types include exonic, intronic, intergenic, UTR3, UTR5, ncRNA_intronic, ncRNA_exonic, and splicing, while the 3 repeat-region types are ALU, REP, and NONREP.

### Curation of TCGA multi-omic data

We provide 13 types of multi-omic data to relate the detected editing events with molecular features of 33 cancers in TCGA and its follow-up studies. From PanCanAtlas 29, we download somatic mutation (2,979,333 mutations), CNV (24,205 genes), 450K beta value of DNA methylation (396,066 CpG sites), tumor purity and ploidy, TPM-normalized gene expression (20,531 genes), miRNA expression (2,455 miRNA), and clinical outcome indices across 33 cancers of TCGA. From 30, we obtain the expression information about 198 proteins of 7,746 TCGA patients. From TIMER2.0 31, we collect the data of immune cell component, which includes the infiltration level of 26 immune cells in 11,010 TCGA tumor samples. From 32, we obtain the data about six tumor stemness indices (DMPsi, ENHsi, mDNAsi, EREG-mDNAsi, mRNAsi, and EREG-mRNAsi) for the measurement of oncogenic dedifferentiation. From 33, we collect the PSI values of five AS types (exon skipping, alternative 3' splice site, alternative 5' splice site, mutually exclusive exons, and intron retention) in 8,705 patients. From 34 and 35, we obtain, respectively, the telomere length and microsatellite instability information. This multi-omic data is summarized as Table 1.

Particularly for gene expression, previous studies had demonstrated gene pathways are essential to the development of cancers, with some being prognostic markers 36. We thus collect 22 pathways commonly shared by cancers. From 36, we obtain 10 canonical signaling pathways across 9,125 samples in 33 cancers, including cell cycle, Hippo, Notch, and P53. Other pathways collected include feeroptosis 37, hypoxia 38, and m6A methylation 39. The gene sets of these 22 pathways are downloaded from MSigDB (https://www.gsea-msigdb.org/gsea/msigdb). Throughout the paper, we use patient ID as our unique index for all data used in the interactive analysis.

### Identification for novel peptides

**Figure 2:**
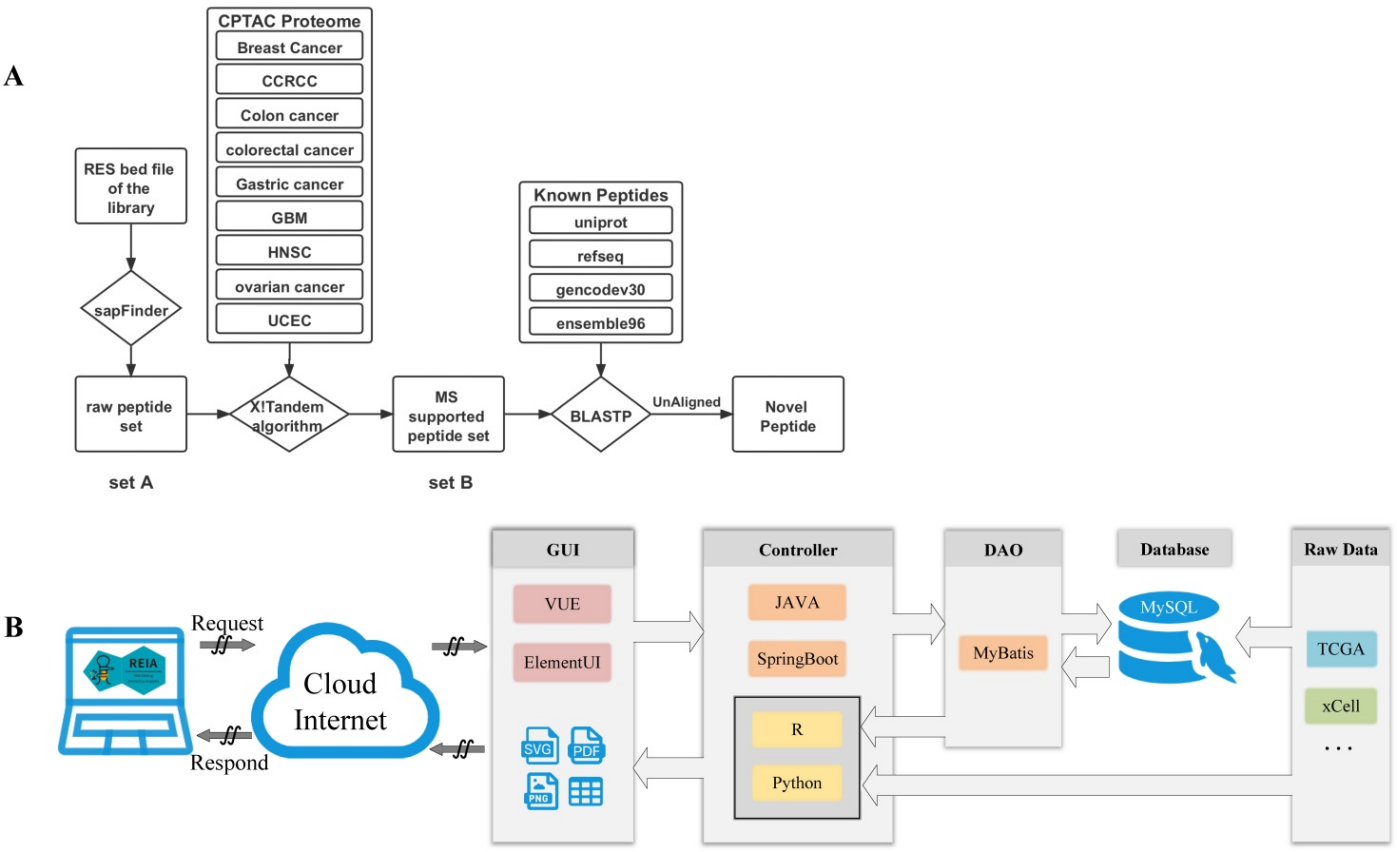
** Data flow and software architecture of the web server REIA. (A)** Detailed pipeline for novel peptide detection. **(B)** Software architecture of the web server.

We also apply a sample-customized search strategy, as shown in Figure 2A, to identify novel peptides that are derived from the detected editing sites.

1) *Construct a set of variation-associated peptides:* First, we generate a variant call format (VCF) file according to the RNA editing sites obtained by using SAMtools and R scripts. Then, we download an mRNA sequence file (GRCh38) and a gene annotation file from UCSC (http://genome.ucsc.edu/cgi-bin/hgTables?command=start). Also, we download the external cross reference file, xref, from MartView (http://biomart.intogen.org/biomart/martview/). Finally, we use the R package of sapFinder 40 (https://academic.oup.com/bioinformatics/article/30/21/3136/2422150) to construct a data set for peptides associated with then RNA editing. A list of all potential peptides resulting from RNA editing events is then obtained and labeled as “peptide set A”.

2) *Select peptides supported by cancer mass spectrum:* In this step, the peptides are validated via mass spectrum (MS) data. We download the mzML format data about 9 cancers from Clinical Proteomic Tumor Analysis Consortium (CPTAC) 41, and extract only the MS2 spectra from each dataset. We then merge the data into a single file in mgf-format using the tool of msconvert from ProteoWizard. After that, we pick out those peptides supported by the MS data via the use of the X!Tandem algorithm 40. That algorithm was particularly useful to the handling of variant identification in high false positive cases. Similar to the prior work of 27, we set the parent ion mass tolerance and fragment ion mass tolerance (monoiso-topic mass) in this study at 10 ppm and 0.1 Da, respectively. Moreover, the protein cleavage site is fixed at ''[KR]j[X]'' to allow for 2 missed cleavages, while the PSM FDR in sapFinder is set to be 0.01. Under the above settings, we have identified 15,035 MS-supported peptides, which are denoted as “peptide set B”.

3)* Identify novel peptides:* Finally, we identify peptides listed in the above “set B” but not yet covered in any of the following databases: Uniprot 42, RefSeq 43, GENCODE V30 44, and Ensembl 96 (https://asia.ensembl.org/index.html). To this end, we apply the Basic Local Alignment Search Tool of Protein Database (BLASTP) to search for peptides that contain at least one unmatching with the peptides of the above databases. We obtain a list of 658 such unmatched peptides and label them as “novel peptides”.

### Implementations of web server

We develop REIA, a new web server for the RNA A-to-I editing interactive analysis, using the MVC framework. MVC is a state-of-the-art design pattern that implements data, user interface, and controlling logic of a software in a divide-and-conquer fashion. Three components are included in MVC: the Model for managing data and business logic, the View for handling layout and display, and the Controller for routing commands to the former two. In Figure 2B, we illustrate the operation flow of our MVC-based web server REIA. Here the View (graphical user interface, GUI) contains a VUE progressive javascript framework and an Element-UI framework. Once a request is received, it forwards that request to the Controller via ajax. The Controller, implemented in a JAVA SpringBoot framework, routes the request the Model which later applies business logic to address the request. In the Model, a DAO-based MyBatis persistence layer framework is used to read data from the MySQL database. This data is then returned to the Controller and gets further processed by R and Python scripts. For figures and tables generated, the Controller decides which View to call and via what display method (supported methods: PDF, PNG, and CSV). It is worthy of noting, in order to reduce the storage burden of the web server, we do not put all data in its online MySQL database. Instead, we leave some data less frequently used to another server in back office. When functions like AS and Methylation are called, such data will be sent directly from the back-office server to the Controller of the (online) server.

## Results and Discussion

### Overview

**Figure 3:**
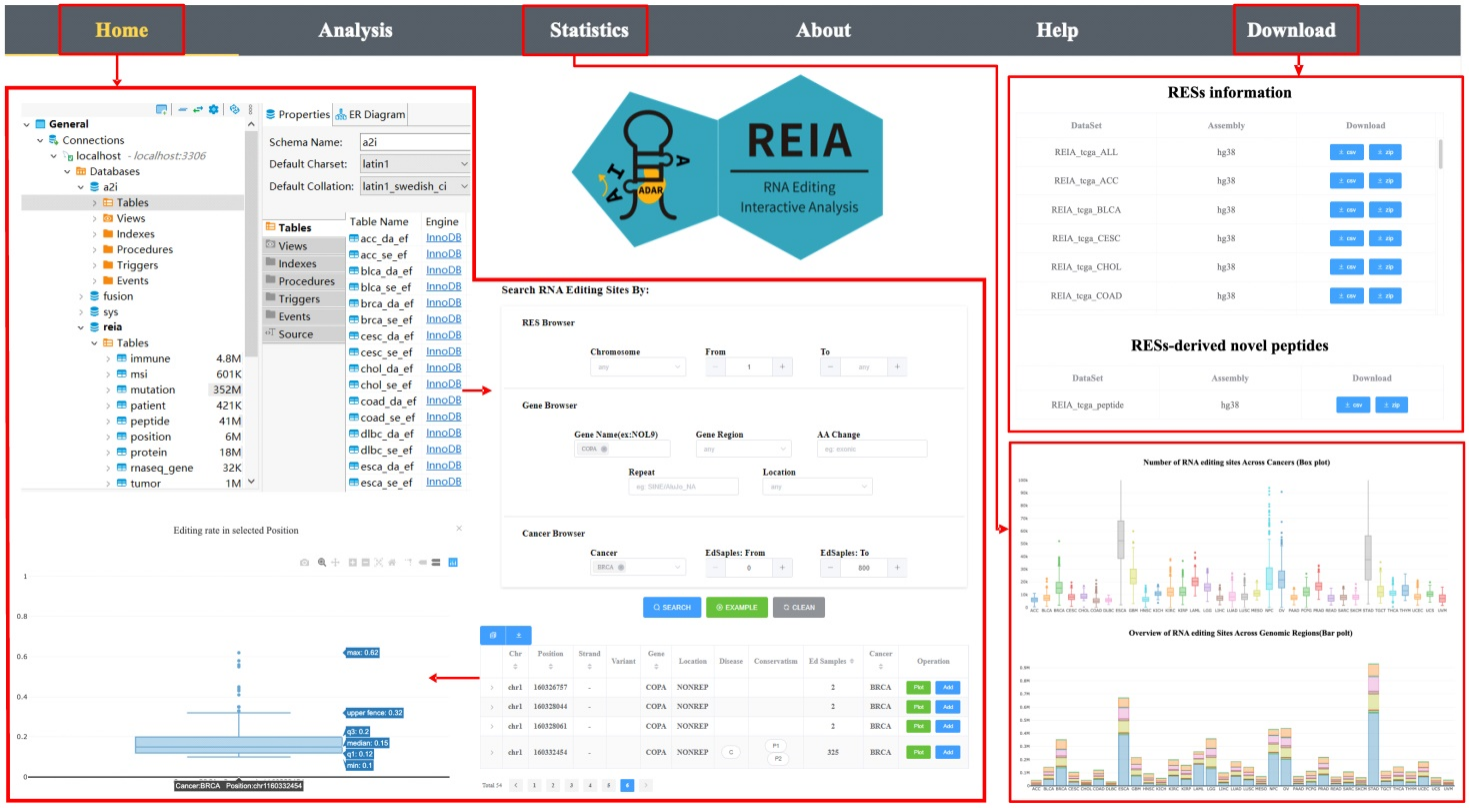
** User interface of the web server REIA.** The navigation page contains 6 tabs: Home, Analysis, Statistics, About, Help, and Download. The “Home” tab retrieves the editing sites and displays their annotation information and editing levels. The “Statistics” tab summarizes the data collected in REIA. The “Download” tab provides links to download the collected data. The “Analysis” tab analyzes the association between RESs and multi-omic features, and this will be introduced later in next figure using an example of the *COPA* I164V editing site.

The web site of REIA has 6 tabs, namely, Home, Analysis, Statistics, About, Help, and Download, where 1) Home: overview of the server, search and display of interested RESs; 2) Analysis: association between RESs and multi-omic molecular features, identification of novel peptides; 3) Statistics: statistical information of the database; 4) About: introduction to the server; 5) Help: usage and examples; 6) Download: data of the database, see Figure 3.

To better demonstrate the usage of “Home” and “Analysis”, we take *COPA* I164V (chr1_160332454) as our example throughout this section. We use *COPA* I164V simply due to its great generality in cancers and its tight connection with other omics features, e.g., patient survival, protein diversity, and gene expression 21, 45. Thus, we pinpoint, in the “Home” page, *COPA* I164V as our target position and move on to the “Analysis” page to divide the patients into two groups (edited or not) and further carry out their interactive analysis with 13 multi-omic features. More details will be elaborated immediately in the two subsections that follow.

### Search and display of editing sites

REIA has collected 8,447,588 editing sites from 34 cancer types for search and display. Among the 34 cancers, STAD, NPC, and ESCA have the highest number of editing sites, while LGG, OV, and BRCA are the second highest (Figure 3, the box plot of “Statistics” page). This difference is partially due to the variation in sample size and/or sequencing depth. Among the 8,447,588 sites, most of the editing sites are located in 3'UTR and intronic (Figure 3, the bar plot of “Statistics” page), which resembles the prior works of 14, 17, 26.

For search, REIA offers 3 inquiry modes in the “Home” page: i) “RESs Browser” for inquiry via RESs, including chromosome and editing site coordinate; ii) “Gene Browser” for inquiry via genes, including gene name, gene region, repeat type, and amino acids change; and, iii) “Cancer Browser” for inquiry via cancers, including cancer type and number of edited samples. Across the three, all items could be combined arbitrarily. To display the search result, REIA provides a table for the annotation and the distribution of each editing site, which contains coordinates, strand, genomic position, reference nucleotide, edited nucleotide, region of cytoband, gene name, gene region, repeated element (if any), potential amino acid change, disease-specific sites, PhyloP and PhastCons conservation score across 100 vertebrates, databases (ATLAS/RADAR/DARNED/TCEA/REDIportal) reporting the RES, and number of edited samples per cancer type. Following each item of the table is a button of “Plot”, which plots the distribution of editing level (defined as the number of edited G reads over the number of A+G reads) at the selected position in each cancer type. Yet another button is “Add”, which adds the current position into the input list of a multi-omic analysis to be detailed in next tab “Analysis”. For the aforementioned example of *COPA* I164V, we use the setup illustrated in Figure 3.

### Interactive analysis with multi-omic data

In the “Analysis” tab, REIA provides, for 33 TCGA cancers, 13 types of interactive analysis at 4 different levels, including DNA, transcriptome, protein, and clinicopathology. The site position(s) of interest can be either imported from the search result (in last tab) or customized at will. Also, all interactive analysis supports the selection of any cancer(s) within the 33. For each selection, the samples are divided into two groups, an “editing” group and a “non-editing”, according to their status at the site position(s). In the analysis that follows, sample ID is used as the unique index for all data. In the same tab, REIA further provides 658 cancer-specific peptides that are derived from A-to-I RNA editing and supported by mass spectrum, but not reported in the existing databases. For each of the analysis functions, the selected index is then plotted for comparison between the two groups. Wilcoxon rank-sum test is adopted in all significance analysis throughout this paper. The p-values here are two-tailed, and Benjamini and Hochberg (BH) FDR is used as a correction for multiple comparisons.

For better illustration of REIA, we take the editing site of *COPA* I164V (chr1_160332454) in breast cancer (BRCA) as our running example. Previous study has shown that the edited *COPA* I164V not only enhances cell viability, wounding healing, migration and invasion significantly, but also make a notable contribution to the tumor development 21.

#### DNA level interactive analysis

As RNA editing affects a variety of molecular features, REIA here investigates 6 major ones on the genome level, i.e., mutation, CNV, telomere length, DNA methylation, MSI, tumor purity and ploidy.

**Figure 4:**
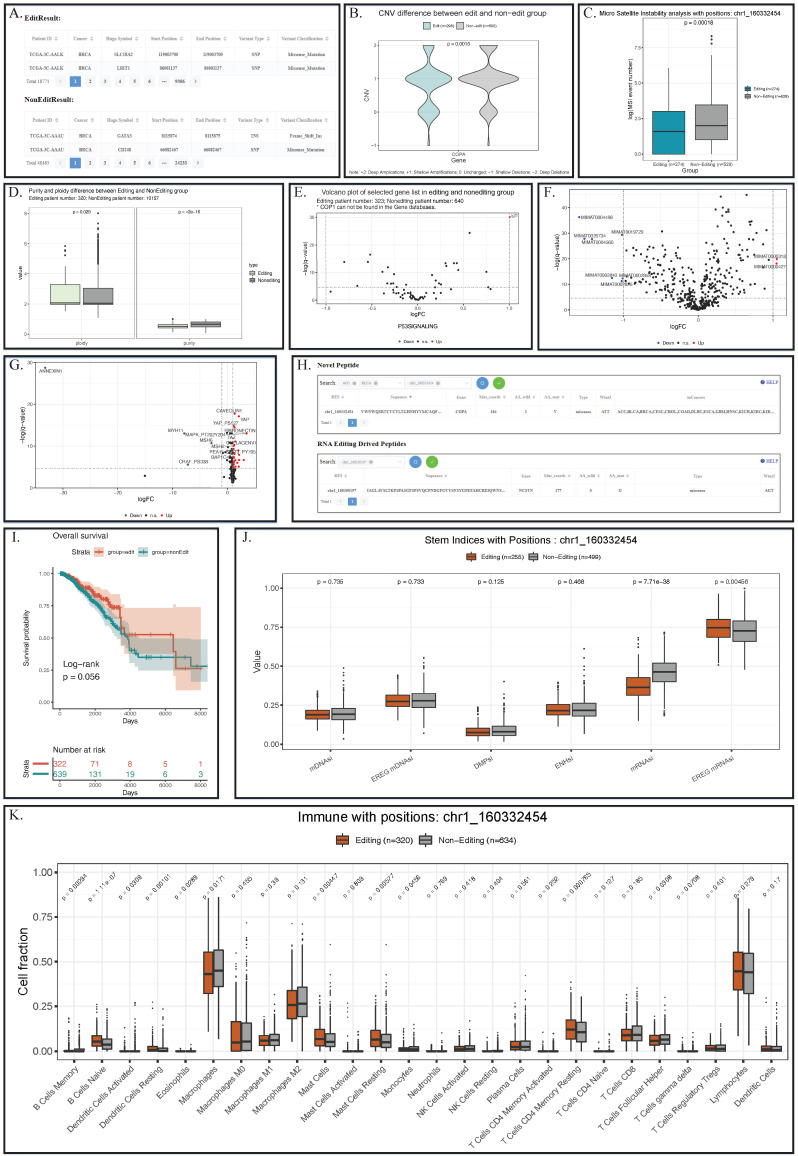
** Analysis result of editing and non-editing groups in the example of *COPA* I164V in BRCA. (A)** Somatic mutation results indicated some mutations that appear specifically in patients with *COPA* I164V event in BRCA. **(B)** Violin plots showing the CNV levels of *COPA* between *COPA* I164V editing and non-editing group across BRCA. **(C)** Boxplot of microsatellite instability between editing and non-editing group patients in BRCA. **(D)** Barplots of Tumor purity and ploidy in the patient group with and without *COPA* I164V event in BRCA. **(E)** Volcano plot indicated that the expression levels of IGF-1 in the P53 signaling pathway of the editing group patients were significantly higher than those in the non-editing group. **(F)** Landscape of differentially expressed miRNA between BRCA editing and non-editing group patients. **(G)** Landscape of differentially expressed proteins between *COPA* I164V editing and non-editing group in BRCA. **(H)** Novel peptides resulted from *COPA* I164V. **(I)** Survival analysis comparing the overall survival of BRCA patients with and without *COPA* I164V editing events. **(J)** Boxplot of six stem indices between two group patients. **(K)** Barplots showing differentially infiltrated immune cells between editing and non-editing group in BRCA.

For somatic mutation, REIA provides 2,017,901 mutation sites. On one hand, somatic mutation is closely related to RNA editing at certain sites 18; on the other hand, RNA editing techniques can repair the somatic mutation of human 13, 46 and even correct the carcinogenic mutation for cancer prevention 13, 47. Despite that, our knowledge of the underlying association between RNA editing and somatic mutation is still fragmental. Now with REIA, one is able to see the difference in such mutation between the editing and non-editing groups. REIA offers two perspectives of somatic mutation analysis, namely, exclusive mutations and enriched mutations. For exclusive mutations, REIA provides a table for the somatic mutations of the editing group and a table for the non-editing group. For enriched mutations, REIA calculates the enrichment p-value for each mutation in the editing group identified by Fisher's exact test. Such analysis allows the users to connect the editing events with mutation profiles and generate various hypotheses on the connections. For example, using REIA, one can identify 18,771 mutations from BRCA patients that have the editing at* COPA* I164V (chr1_160332454). For each somatic mutation, one can further check information like its overlapped gene, genome coordinate position, and the variant classification (Figure 4A).

For CNV, REIA offers the information of 24,205 variations. CNVs is known to influence the cancer's global abundance of protein and phosphosite 48, 49; however, its connection with RNA A-to-I editing is still not clear. Here, REIA provides an opportunity to explore such potential connections in a quantitative way. To be specific, one can pin point the interested editing site(s), gene name(s), and/or cancer type(s), and obtain a violin plot that quantify the CNV difference between the editing and the non-editing groups. In the example of *COPA* I164V, the editing group exhibits a level of copy number amplification significantly higher than the non-editing group (p=0.0015, Figure 4B).

For telomere length, REIA compares the distributions of the editing and the non-editing groups. Stability of telomere tandem repeats (TTAGGG)n hexameric DNA repeats of telomeres) is critical to cancer progression, as it ensures both the stability of chromosomes and the integrity of genome: the shorter a telomere, the higher its risk in cancer 50. In this context, REIA offers a comparison in boxplot-based telomere length between the editing and the non-editing groups. Such comparison facilitates the studies on possible associations between RNA editing and telomere length.

For DNA methylation, REIA calculates the β values therein. As the prior work demonstrated 51, DNA methylation directly affects microRNA biogenesis in mammalian cells, thus resembling the RNA editing in many aspects. However, its association with RNA editing is yet to be verified. For this reason, REIA provides volcano plot-based DNA methylation comparison between the patients with and without RNA editing. The comparison can serve as a starting point for the association analysis between RNA editing and DNA methylation.

For microsatellite instability (MSI), REIA plots the distribution of its event number. MSI, as a major carcinogenetic pathway 52, has a distribution recognized as the implication for many cancers 53. For that reason, REIA implements the comparison of MSI indices between the editing and the non-editing groups, which may help to identify clinical targets. In the *COPA* I164V example, the MSI in the editing group is seen to be significantly higher than the non-editing group (p=0.00018, Figure 4C).

For tumor purity and ploidy, REIA computes the two values to investigate their (possible) association with the A-to-I editing. Tumor purity and ploidy have a great impact on cancer genomic evolution and tumor heterogeneity, thus affecting severely cancer progression and patient survival 54, 55. According to the boxplot, a significantly higher level of ploidy other than purity could be observed in the patient with chr1_160332454 editing in BRCA (p=0.029 and p=2e-16, Figure 4D).

#### Transcript level interactive analysis

RNA editing in cancers may affect many features on the transcript level 56. In REIA, we analyze 3 of these aspects, namely, gene expression, alternative splicing, and microRNA expression.

REIA provides 20,531 gene expressions and the gene sets of 22 classic pathways related to cancers. A-to-I RNA editing was known to have an overall influence on the gene expression of most cancers 36. Here REIA offers an approach to precisely measure the difference between the editing and non-editing groups on the expression of a single gene or gene set. With the boxplot REIA generated, the users can explore the underlying influence of editing events on a single gene. With the volcano plot generated, users can determine the differentially expressed genes in any gene sets. In the *COPA* I164V example, the expression level of *IGF-1* (insulin-like growth factors) in the P53 signaling pathway of the editing group can be seen to be significantly higher than that of the non-editing group (Figure 4E, the cutoffs for log2FC and p-value are 1 and 0.01, respectively). Note that the over-expression of this gene had long been known in cancers like GBM and HCC.

REIA computes the PSI values for five alternative splicing forms, namely, exon skipping, alternative 3' splice site, alternative 5' splice site, mutually exclusive exons, and intron retention, according to this formula PSI value = splice_in / (splice_in + splice_out). Alternative splicing, a key factor in prognostic analysis, also affects the individual changes in regulatory binding sites and the alterations to protein-coding sequences 57-60, but its association with the RNA editing is not yet explored. With REIA, it is now ready.

REIA has collected 2,455 miRNA expressions from 9,406 patients. As high-throughput detection suggests, the A-to- I RNA editing occurs usually in non-coding RNAs, especially in microRNAs (miRNAs). RNA editing is often found in the binding domain of miRNAs and mRNA 61 and in pri-miRNA 19. About 20% of pri-miRNAs are edited 62, of which more than 550 positions are edited in human context 20. Again, its association with the miRNA expression needs further studies. REIA offering access to the comparison of any miRNA expression about patients with distinct RNA editing profiles. With the boxplot generated by REIA, the users can explore the underlying influence of editing events on a single miRNA. With the volcano plot, the users obtain the miRNA differentially expressed between two groups. The result of the *COPA* I164V example is given in Figure 4F, where the cutoffs for log2FC and p-value are set to be 1 and 0.01, respectively.

#### Protein level interactive analysis

1) REIA provides 197 protein expressions of the TCGA patients. For cancers, A-to-I RNA editing in certain positions, such as I164V in *COPA* (i.e., our example), S367G in *AZIN1*, and I635V in *COG3*, is the root cause of protein expression dysregulation and proteomic diversity 27. Also, the editing in these positions is cross-tumor nonsynonymous and affects the drug sensitivity 21, thus showing clinically relevant patterns in cancers 21, 63. In “Box Plot” of protein expression module, one can compare the difference between the two groups in any cancer(s), any protein(s), or their combinations. In “Volcano Plot”, one can further explore the protein expression with customized thresholds. Such a plot visualizes the (potential) connection between proteins and signaling pathways affected by RNA editing. In the example of *COPA* I164V, the volcano plot presents a series of proteins differentially expressed between the editing and the non-editing groups (Figure 4G, the cutoffs for log2FC and p-value are 1 and 0.01, respectively).

2) REIA also identifies 658 novel peptides, which were derived from the A-to-I RNA editing and supported by MS data from related literature. Although they are cancer specific, these peptides are not yet included in any protein database currently available. For these peptides, REIA offers the RES coordinates, sequence, gene name, mutant and wild-type bases on amino acids, codons, and distribution in cancers, which are essential to target therapy (Figure 4H). It should be noted that, according to the Human Proteome Organization (HUPO, www.hupo.org) guideline, a novel peptide is confirmed by the identification of at least two non-nested peptides covering the residue site with an amino acid change. This aspect is not covered in the current paper and left for further study. However, the peptides identified here are supported by mass spectrum data; hence, they serve well as good starting points for downstream experiments.

#### Clinicopathology level interactive analysis

1) Patient Survival: REIA provides the overall survival and the progression-free survival data of 11,160 patients. As exemplified in 12, effective prognostic models could be built using the information about A-to-I RNA editing sites. REIA provides an approach to analyze the patient's survival affected by any editing site(s). The survival of each group is estimated via the Kaplan-Meier method and compared using the log-rank test. In each test, the survival curves are plotted with 95% confidence interval, and the result of the *COPA* I164V example is shown in Figure 4I.

2) Stem Indices: REIA provides 6 stemness indices summarized from the 9,399 patients. Commonly found in the metastatic tumors, the stemness indices have a significantly higher level of dedifferentiation in cancer progression 64, 65. To tumor stemness RNA editing plays the role of an enhancer, e.g., the AZIN1 RNA editing confers cancer stemness and enhances the oncogenic potential 66. REIA provides an opportunity to look into the impact of RNA editing on six tumor stem indices (DMPsi, ENHsi, EREG-mDNAsi, mDNAsi, EREG-mRNAsi, and mRNAsi). In the example of *COPA* I164V, mRNAsi and EREG-mRNAsi stem indices can be seen to be significantly higher in the editing group, suggesting that the editing is associated with stemness (Figure 4J).

3) Tumor Immune Microenvironment: REIA has collected 26 indices of immune cell infiltration from 11,010 patients. A-to-I RNA editing is known to get evolved in the discrimination of self and nonself RNA; the human RNA editing enzyme ADAR1 prevents endogenous RNA from activating innate immune sensors (PKR, MDA5), thus regulating the delicate balance between pathogen detection and protection versus autoinflammation and disease 67. Also, the upregulated ADAR1 could result in excessive RNA editing, triggering abnormal immune responses and promoting the risk of various cancers 68, including breast, colorectal, and lung 69-71. As an effort to explore immune cells that associate with RNA editing, REIA allows its users to compare the infiltration level of 26 immune cells and to identify immune cells that are differentially present in the editing and non-editing groups. In the *COPA* I164V example, the result shows that 6 immune cells are significantly different (Figure 4K, Wilcoxon rank-sum test, p-value cutoff 0.01).

## Conclusion

The A-to-I RNA editing was repeatedly found involved in cellular functions; however, the biological role of the editing in human cancers has not been fully elucidated. In literatures currently available, databases were mostly for the A- to-I RESs of healthy tissues, lacking in cancer-centric solutions. This paper provides one such solution, the REIA, a database for A-to-I RNA editing in cancers with interactive analysis on the association with multi-omic features. On one hand, REIA has detected 8,447,588 A-to-I RESs from the RNA-Seq data of 9,895 tumor patients across 34 cancers. REIA also collected 13 types of multi-omic data related to human cancers. In addition, it further identified 658 novel peptides from non-synonymous peptides supported by mass spectrum data, which paves the way for targeted therapies. On the other hand, REIA applied MVC to handle user interface, business logic, and data management in a divide-and-conquer fashion, making the interaction and maintenance much easier than prior works. In terms of interactive analysis, REIA not only provides a retrieval function for the editing sites detected, but also analyzes/visualizes their association with multi-omic features. In summary, REIA, as a cancer-centric database, opens up a new avenue for the study of associations between A-to-I RNA editing and multi-omic features.

REIA could be further enhanced in the following aspects. First, genome sequence data can be added to evaluate more fully the influence of nucleic acid sequence alterations on cancers, as we all know editing is a root cause of sequence diversity in cancers 72 (while other causes include DNA mutations and epi-transcriptomic changes). Second, RNA seq data of normal tissues can be analyzed as the control group to single out editing events that are cancer specific. Third, more novel editing sites can be detected either from hyper-edited reads of TCGA cancers or from the RNA-seq data of other cancers in databases like International Cancer Genome Consortium (ICGC, https://icgc.org). Fourth, a new analysis for ncRNA A-to-I RNA editing can be considered. Unlike the extensively studied recording RESs 21, 27, the noncoding RNA editing sites (or ncRNA A-to-I RESs) are relatively less known. These noncoding RNA editing sites, although previously assumed to be deregulated in cancers, are recently used as the clinical biomarkers and thus play an increasingly important role in tumor immunity 3, 73-75. Fifth, single-cell data can be introduced to complement the database. Last but not least, structure of the 658 novel peptides identified can be predicted via AlphaFold2 76. Such structure information may help the understanding of relationship between RNA editing and the changes in protein structure, protein function, and drug targets.

## Key Points

- A new database, REIA (http://bioinfo-sysu.com/reia), has been implemented, providing a comprehensive resource for the analysis of association between A-to-I RNA editing and human cancers.
- For REIA, 8,447,588 editing sites were curated from 9,895 patients across 34 cancer types, among which 33 were from TCGA and 1 from GEO.
- 13 types of multi-omic data related to the cancers were curated manually to perform an association analysis with the editing sites in an interactive fashion.
- A user-friendly interface was implemented for REIA, where the users could browse, search, analyze and download the data of the editing sites and/or association analysis.
- 658 novel peptides were also detected from the editing profiles, all of which were supported by mass spectrum data and thus could serve as clues for downstream experimental design.

## Figures and Tables

**Table 1:** Summary of multi-omic data

Features	Indices	Patients
Somatic mutation	2,979,333	10,225
Copy number variation (CNV)	24,205	9,991
DNA methylation	396,066	9665
Telomere length	1	8,516
Purity and ploidy	2	10,786
Microsatellite instability (MSI)	1	7,920
Gene expression (TPM)	20,531	10,250
miRNA expression	2,455	9,405
Alternative splicing	5	8,705
Protein expression	198	7,746
Immune cell infiltration	26	11,010
Stem indices	6	9,399
Patient Survival	2	11,160

## Abbreviations

BRCA: Breast Invasive Carcinoma
CCRCC: Clear Cell Renal Cell Carcinoma
CML: Chronic Myeloid Leukemia
CNV: Copy Number Variation
CPTAC: Clinical Proteomic Tumor Analysis Consortium
GBM: Glioblastoma multiforme
HNSC: Head and Neck squamous cell carcinoma
MS: Mass Spectrometry
MSI: Microsatellite Instability
TCGA: The Cancer Genome Atlas
THCA: Thyroid Carcinoma
TPM: Transcripts Per Million
UCEC: Uterine Corpus Endometrial Carcinoma
